# Cost-effectiveness of sacituzumab govitecan in hormone receptor-positive/human epidermal growth factor receptor 2-negative metastatic breast cancer

**DOI:** 10.3389/fonc.2023.1162360

**Published:** 2023-05-12

**Authors:** Demin Shi, Yan Li, Xueyan Liang, Lingyuan Chen

**Affiliations:** ^1^ Department of Reproductive Medicine, The People’s Hospital of Hechi, Hechi, Guangxi, China; ^2^ Department of Pharmacy, Guangxi Academy of Medical Sciences and the People’s Hospital of Guangxi Zhuang Autonomous Region, Nanning, Guangxi, China; ^3^ Department of Pharmacy, The People’s Hospital of Hechi, Hechi, Guangxi, China

**Keywords:** cost-effectiveness, sacituzumab govitecan, breast cancer, hormone receptor-positive, human epidermal receptor 2-negative, partitioned survival model

## Abstract

**Background:**

The efficiency and safety of sacituzumab govitecan (SG) for the therapy of hormone receptor-positive (HR+)/human epidermal receptor 2-negative (HER2-) metastatic breast cancer (BC) has been demonstrated. The aim of this study is to evaluate its cost-effectiveness on HR+/HER2- metastatic BC from the third-party payer perspective in the United States.

**Methods:**

We performed the cost-effectiveness of SG and chemotherapy using a partitioned survival model. TROPiCS-02 provided clinical patients for this study. We evaluated the robustness of this study by one-way and probabilistic sensitivity analyses. Subgroup analyses were also conducted. The outcomes were costs, life-years, quality-adjusted life years (QALYs), incremental cost-effectiveness ratio (ICER), incremental net health benefit (INHB), and incremental net monetary benefit (INMB).

**Results:**

SG treatment was related to an increase of 0.284 life years and 0.217 QALYs over chemotherapy, as well as a cost increase of $132,689, reaching an ICER of $612,772/QALY. The INHB was -0.668 QALYs, and the INMB was -$100,208. SG was not cost-effective at the willingness to pay (WTP) threshold of $150,000/QALY. The outcomes were sensitive to patient body weight and cost of SG. SG may be cost-effective at the WTP threshold of $150,000/QALY if the price is less than $3.997/mg or the weight of patients is under 19.88 kg. Based on the subgroup analysis, SG did not prove cost-effective in all subgroups at the WTP threshold of $150,000/QALY.

**Conclusion:**

From a third-party payer standpoint in the United States, SG was not cost-effective, even though it had a clinically significant advantage over chemotherapy for the treatment of HR+/HER2- metastatic BC. The cost-effectiveness of SG can be improved if the price is substantially reduced.

## Introduction

Globally, breast cancer (BC) surpass lung cancer as the most common malignancy diagnosed in 2020, with 2.3 million new cases (1). BC is common cancer in women (24%) and is the leading cause of cancer-related deaths (15%) worldwide (1). The diagnosis of BC was made in approximately 42% of women in the Asia-Pacific region and 47% in Southeastern Asia, as well as 20% of women in Western countries (2, 3). Molecular subtypes of BC have been defined according to the status of hormone receptors (HR), such as estrogen receptor (ER) and progesterone receptor (PR) and human epidermal growth factor 2 (HER2) (4). Approximately 70% of cases of BC are classified as luminal, a molecular subtype characterized by HR-positive (HR+) and HER2-negative (HER2-). Endocrine therapy (ET), which covers aromatase inhibitors (AIs), selective ER modulators (SERMs), and selective ER down-regulators (SERDs), forms the foundation for the effective treatment of BC (5–8). In the absence of ET resistance, either primary or secondary, subsequent treatment options are limited; there are only a few therapy options available for premenopausal women with HR+/HER2- metastatic BC, and these are mostly derived from trials in which postmenopausal patients were enrolled (9). By combining endocrine therapy with CDK4/6 inhibitors (CDK4/6i), overall survival (OS) for HR+/HER2- metastatic BC can be improved by approximately five years (10–13). In subsequent treatment lines, combination therapy with phosphoinositide 3-kinase inhibitors or mammalian target of rapamycin inhibitors has been shown to be beneficial (8). It is inevitable, however, that endocrine resistance will develop over time. The next therapeutic option is sequential single-agent chemotherapy, but it has declining response rates, diminished disease control, and related to high risk of side effects (8, 14–17).

Sacituzumab govitecan (SG) is a first-in-class antibody-drug conjugate directed at trophoblast cell-surface antigen 2 (Trop-2) consisting of a humanized polyclonal antibody conjugated to the active metabolite SN-38 (18), by a hydrolysable CL2A linker (19, 20). In solid tumors, particularly HR+/HER2- and triple-negative breast cancers (suffering from a prevalence of > 90%), Trop-2 is a transmembrane calcium signal transducer that is associated with tumor progression and prognosis (21, 22). In tumor microenvironments, SN-38 is a membrane-permeable free molecule that may exert antitumor effects on tissues adjacent to those that do not express Trop-2 (bystander effect) (23). As SG was shown to be clinical beneficial and safety in patients with HR+/HER2- metastatic BC who had progressed after completing endocrine therapy and prior chemotherapy in the metastatic setting, the results were encouraging (24, 25). There was, however, a significant increase in the cost of SG treatment, which may limit its availability in some countries (26). SG has not yet been evaluated on an economic basis for its use in treating HR+/HER2- metastatic BC. It is essential for clinicians and policy-makers to consider cost-effectiveness when making healthcare decisions. Herein, cost-effectiveness analysis of SG in comparison with single-agent chemotherapy for HR+/HER2- metastatic BC was conducted from the perspective of third-party payers in the United States.

## Methods

### Analytical overview

This analysis was conducted on hypothetical patients who had locally recurrent, metastatic HR+/HER2- BC that was endocrine-resistant and treated with chemotherapy, included HR+/HER2- metastatic BC patients from the TROPiCS-02 trial (25). The economic evaluation used a partitioned survival model with three health states to determine whether to use SG or single-agent chemotherapy for the initial treatment decision (27–30). Progression-free survival (PFS), progressed disease (PD), and death are mutually exclusive health states. The area under the OS curve was used to estimate the proportion of patients alive at cycle t (1-week cycle), and the area under the PFS curve was used to estimate the proportion of patients alive with PFS. Based on the difference between the OS and PFS curves, the proportion of patients alive and suffering from PD was estimated. The patients and PFS and OS curve were derived from the TROPiCS-02 trial (25), whose results were validated by comparing modeled PFS and OS to real data. We performed this study following the reporting guideline of Consolidated Health Economic Evaluation Reporting Standards (CHEERS) (31). In view of the fact that this study used a review of publicly available data and modeling techniques, it will not require an institutional review board review or informed consent.

### Clinical data inputs

TROPiCS-02 results were obtained to construct PFS and OS for patients in the SG and chemotherapy groups (24) and the data have been extrapolated beyond the follow up time of the model using the statistical methods described by Guyot et al (32). To collect the time-to-survival data points from the PFS and OS curves, we utilized the GetData Graph Digitizer, version 2.26 (33), and the following parametric survival functions were then fitted to these data points: exponential, Weibull, gamma, lognormal, Gompertz, Log-logistic and Generalized gamma models. It was determined that the eligible survival function had the lowest Akaike information criterion and Bayesian information criterion values. SG treatment and chemotherapy treatment final survival functions are illustrated in **Table 1**, as well as goodness-of-fit results were shown in **Supplementary Table 1**. PFS and OS proportions were calculated based on the appropriate survival distribution. Model validations are shown in **Supplementary Figure 1**. A digitized Kaplan-Meier curve was closely reproduced in the virtual patient-level data, which included event and censoring times.

**Table 1:** Basic parameters input to the model and the ranges of the sensitivity analyses.

Parameter	Value (95% CI)	Distribution	Source
**Clinical input**			
**Survival model for sacituzumab govitecan**			
Log-logistic model for OS^a^	γ = 1.9025; λ = 0.0162	ND	(25)
Log-normal model for PFS^a^	μ = 3.1013; σ = 1.0541	ND	(25)
**Survival model for chemotherapy**			
Log-logistic model for OS^a^	γ = 1.9082; λ = 0.0188	ND	(25)
Log-normal model for PFS^a^	μ = 2.7297; σ = 0.9475	ND	(25)
**Cost input**			
**Drug costs per 1 mg**			
Sacituzumab govitecan	14.88 (11.91 to 17.86)	Gamma	(34)
Eribulin	1266 (1013 to 1520)	Gamma	(35)
Vinorelbine	0.925 (0.740 to 1.110)	Gamma	(35)
Gemcitabine	0.018 (0.014 to 0.021)	Gamma	(35)
Capecitabine	0.004 (0.003 to 0.005)	Gamma	(35)
Cost of terminal care per patient^b^	21,501 (17,201 to 25,801)	Gamma	(36)
**Disease management and monitoring costs**			
CT scan of chest (per time)	133 (58 to 254)	Gamma	(37)
Best supportive care (per cycle)	472 (377 to 566)	Gamma	(38)
Cost of managing AEs (grade ≥ 3)^c^			
Sacituzumab govitecan	7,309 (5,847 to 8,770)	Gamma	(39–41)
Chemotherapy	5,287 (4,230 to 6,344)	Gamma	(39–41)
**Administration cost**			
First hour	159 (130 to 206)	Gamma	(37)
Additional hour	34 (28 to 42)	Gamma	(37)
**Health utilities**			
**Disease status utility per year**			
PFS	0.830 (0.664 to 0.935)	Beta	(39, 42)
PD	0.443 (0.354 to 0.532)	Beta	(39, 43)
Death	0	NA	
Disutility due to AEs^d^			
Sacituzumab govitecan	0.037 (0.03 to 0.044)	Beta	(39–41)
Chemotherapy	0.023 (0.018 to 0.027)	Beta	(39–41)
**Other inputs**			
Body surface area, m^2^	1.82 (1.44 to 2.16)	Normal	(44)
Body weight, kg	74 (59 to 90)	Normal	(44)

AE, adverse event; NA, not applicable; ND, not determined; OS, overall survival; PD, progressed disease; PFS, progression-free survival.

a Only expected values are presented for these survival model parameters.

b Overall total cost per patient regardless of treatment duration.

c Calculated as the average cost of toxic effects using weighted frequencies of grade ≥ 3 treatment related adverse events for each treatment arm in the TROPiCS-02 trial. Costs of individual toxic effects were derived from the literature and include all care required to manage each toxic effect. References for individual toxic effect costs are summarized in **Table 2** in the Supplement.

d Calculated as the average disutility of toxic effects using weighted frequencies of grade ≥ 3 treatment-related adverse events for each treatment arm in the TROPiCS-02 trial. Disutilities of individual toxic effects were derived from the literature. References for individual toxic effect disutilities are summarized in **Table 2** in the Supplement.

### Cost and utility inputs

In this study, we evaluated the costs related to direct medical costs, covering the costs of drugs, the costs associated with terminal care, the costs related to the management of patients, and the costs related to adverse events (AEs) (**Table 1**). The costs are reported in 2023 United States dollars and other costs have been inflated using Tom’s Inflation Calculator’s Medical Care Inflation set (45).

In the TROPiCS-02 trial report (25), patients received SG 10 mg/kg body weight intravenously on days 1 and 8 of every 21 days. The treatment was continued until the disease progressed or the side effects became unacceptable. It is expected that patients assigned to the chemotherapy group received treatment according to locally approved prescribing information or according to National Comprehensive Cancer Network Clinical Practice Guidelines in Oncology (46). Recommended chemotherapy regimens dosage of TROPiCS-02 are following: eribulin, 1.4 mg/m^2^ for North American or 1.23 mg/m^2^ for European; vinorelbine, 25 mg/m^2^; gemcitabine, 200 mg/m^2^; and capecitabine 1,000-1,250 mg/m^2^ (24).

The prices of SG, eribulin, vinorelbine, gemcitabine and capecitabine were collected from public databases (34, 35). The cost of terminal care was $21,501 per patient with metastatic BC (36). The cost of the CT scans was obtained from the Medicare Clinical Laboratory Fee Schedule (37). The costs of the best supportive care were $472 per cycle (38). This study included the costs of managing grade ≥ 3 AEs, which were obtained from the published literature (**Supplementary Table 2**) (39–41). To calculate the dosage of SG, eribulin, vinorelbine, gemcitabine and capecitabine, we assumed that the body weight and body surface area of a typical patient in the United States were 74 kg and 1.82 m^2^ (44).

Health states were rated on a scale of 0 to 1 according to their utility preference in terms of health. Considering TROPiCS-02 was not provided the results of utility, the utility of metastatic BC was obtained from previously published studies and the PFS and PD states related to metastatic BC were 0.830 and 0.443 respectively (39, 42, 43). The analysis evaluated the disutility values related to grade ≥ 3 AEs (39–41).

### Base-case analysis

We calculated the incremental cost-effectiveness ratio (ICER) by comparing the incremental cost per quality-adjusted life year (QALY) gained between the SG group and the chemotherapy group. According to the recommendation, cost-effectiveness was assumed when the ICER was lower than the optional willingness to pay (WTP) threshold ($150,000 per additional QALY gained) (47). Costs and utilities were discounted at an annual rate of 3% (48). We calculated the incremental net health benefit (INHB) and incremental monetary benefit (INMB) using the following formulas: $INHB\left(\lambda \right)=\left(\mu E_{SG}-\mu E_{c}\right)-\frac{\mu C_{SG}-\mu C_{C}}{\lambda }=\;\Delta E-\Delta C/\lambda$ and $INMB\left(\lambda \right)=\left(\mu E_{SG}-\mu E_{c}\right)\times \lambda -(\mu C_{SG}-\mu C_{C})=\;\Delta E\times \lambda -\Delta C$ , where μC_SG_, μC_C_, and μE_SG_, μE_C_ were the cost and QALY of SG or chemotherapy, respectively, and λ was the WTP threshold (49, 50).

### Sensitivity and subgroup analyses

Based on the one-way sensitivity analysis and the probabilistic sensitivity analysis, we evaluated the robustness of the model results. Each parameter was subjected to a one-way sensitivity analysis; estimated ranges were based on the reported or estimated 95% confidence intervals in the referenced studies or assumed to change 25% from the base-case value (**Table 1**). In order to generate a probabilistic sensitivity analysis, the key model variables were simultaneously sampled from prespecified distributions in a Monte Carlo simulation with 10,000 iterations. A gamma distribution was used for the cost variables, and a beta distribution for was used probability and proportion. To calculate the likelihood that SG would consider being cost-effective at different WTP levels, a cost-effectiveness acceptability curve was constructed based on data from 10,000 iterations. Subgroup analyses were conducted by varying the HRs for PFS for the prespecified subgroups reported in TROPiCS-02 in order to investigate the uncertainty arising from the subpopulations. We conducted our statistical analyses in R, version 4.0.5, 2021 (R Foundation for Statistical Computing) using the hesim and heemod packages.

## Results

### Base-case analysis

By comparison with chemotherapy treatment, SG treatment increased QALYs by 0.217 and overall life-years by 0.284, at an incremental cost of $132,689, which corresponds to a QALY ICER of $612,772. The INHB was -0.668 QALYs, and the INMB was -$100,208 at a WTP threshold of $150,000/QALY (**Table 2**).

**Table 2:** Summary of cost and outcome results in the base-case analysis.

Factor	Sacituzumab govitecan	Chemotherapy	Incremental change
**Cost, $**			
Drug^a^	139,829	13,267	126,562
Nondrug^b^	57,552	51,425	6,127
Overall	197,381	64,692	132,689
**Life-years**			
Progression-free	0.737	0.451	0.286
Overall	1.766	1.482	0.284
**QALYs**			
Progression-free	0.592	0.368	0.224
Overall	1.016	0.799	0.217
**ICERs, $**			
Per life-year	NA	NA	467,013
Per QALY	NA	NA	612,772
INHB, QALY, at WTP threshold 150,000^a^	NA	NA	-0.668
INMB, $, at WTP threshold 150,000^a^	NA	NA	-100,208

ICER, incremental cost-effectiveness ratio; INHB, incremental net health benefit; INMB, incremental net monetary benefit; NA, not applicable; QALYs, quality-adjusted life years.

a Compared with chemotherapy.

b Nondrug cost includes the costs of adverse event management, subsequent best supportive care per patient, and follow-up care covering physician monitors, drug administration, and terminal care.

### Sensitivity analysis

One-way sensitivity analyses suggested that the HRs for OS, average body weight, HRs for PFS and the costs of SG were related to model results (**Supplementary Figure 2**). We also estimated the relationship between these key variables and the ICER in the comparison of SG and chemotherapy. When the price of SG was less than $2.821/mg or $3.997/mg, SG was cost-effective at a WTP threshold of $100,000/QALY or $150,000/QALY, respectively (**Supplementary Figure 3**). On the other hand, when the body weight of patients was less than 19.88 kg, SG was cost-effective at a WTP threshold of $150,000/QALY (**Supplementary Figure 3**).

**Figure 1:**
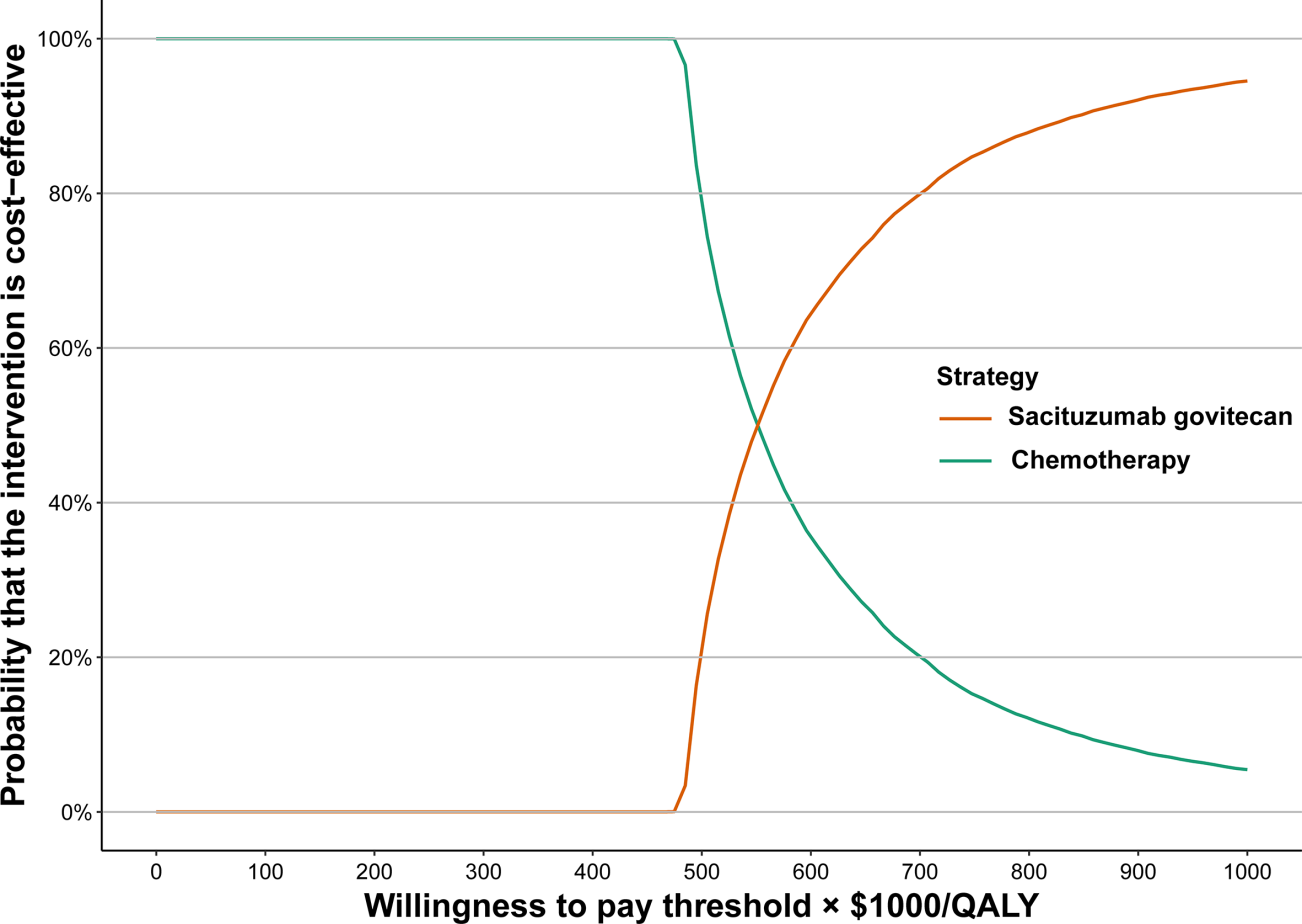
Acceptability curves for the choice of sacituzumab govitecan treatment strategies at different WTP thresholds in patients with hormone receptor-positive/human epidermal growth factor receptor 2-negative metastatic breast cancer. WTP, willingness to pay.

The cost-effectiveness acceptability curve was calculated and displayed as a result of the probabilistic sensitivity analysis (**Figure 1**). When the WTP thresholds are raised, the probability of SG being cost-effective increases. In comparison with chemotherapy, SG had no probability of being considered cost-effective at a WTP threshold of $150,000/QALY.

### Subgroup analysis

By varying the HRs for PFS, the subgroup analyses suggested that SG was related to primarily negative INHBs and was not considered cost-effective at a WTP threshold of $150,000/QALY for all subgroups (**Table 3**).

**Table 3:** Summary of subgroup analyses obtained by varying the hazard ratios (HRs) for PFS.

Subgroup	Unstratified hazard ratio (95% CI)	Change in cost, $^a^	Change in QALYs^a^	ICER, $/QALY	INHB, QALY, at WTP threshold 150,000
**Visceral metastasis**					
Yes	0.66 (0.53 to 0.83)	132,689	0.217	612,772	-0.668
No	0.78 (0.25 to 2.40)	131,978	0.182	724,906	-0.698
**Endocrine therapy in the metastatic setting ≥ 6 months**					
Yes	0.61 (0.48 to 0.78)	133,024	0.230	578,048	-0.657
No	1.13 (0.61 to 2.07)	130,329	0.067	1,938,885	-0.802
**Age, years**					
<65	0.69 (0.53 to 0.89)	132,500	0.208	636,506	-0.675
≥ 65	0.59 (0.38 to 0.93)	133,167	0.235	565,617	-0.652
**Race**					
White	0.66 (0.51 to 0.86)	132,689	0.217	612,772	-0.668
Non-white	1.23 (0.55 to 2.75)	129,922	0.031	4,172,372	-0.835
**Baseline ECOG performance status scale score**					
0	0.61 (0.44 to 0.86)	133,024	0.230	578,048	-0.657
1	0.70 (0.53 to 0.94)	132,439	0.205	644,971	-0.678
**Geographic region**					
North America	0.72 (0.51 to 1.02)	132,319	0.200	662,817	-0.682
Europe	0.62 (0.46 to 0.82)	132,955	0.227	584,559	-0.659
**Prior CDK inhibitor duration**					
≤ 12 months	0.59 (0.44 to 0.78)	133,167	0.235	565,617	-0.652
> 12 months	0.77 (0.54 to 1.10)	132,033	0.185	713,553	-0.695
**Investigator choice of chemotherapy**					
Eribulin	0.71 (0.55 to 0.93)	116,558	0.202	575,610	-0.575
Capecitabine	0.91 (0.53 to 1.57)	144,849	0.142	1,021,786	-0.824
Gemcitabine	0.83 (0.54 to 1.28)	144,964	0.167	868,473	-0.800
Vinorelbine	0.32 (0.22 to 0.47)	144,238	0.301	479,550	-0.661
**Early relapse**					
Yes	0.10 (0.04 to 0.28)	140,665	0.349	403,091	-0.589
No	0.72 (0.57 to 0.91)	132,319	0.200	662,817	-0.682
**No. of prior chemotherapy in metastatic setting**					
≤ 2	0.62 (0.45 to 0.85)	132,955	0.227	584,559	-0.659
≥ 3	0.70 (0.52 to 0.95)	132,439	0.205	644,971	-0.678

CDK, cyclin-dependent kinase; ECOG, Eastern Cooperative Oncology Group; HR, hazard ratio; ICER, incremental cost-effectiveness ratio; INHB, incremental net health benefits; PFS, progression-free survival; QALY, quality-adjusted life year; WTP, willingness to pay.

a HR for PFS represents the HR of sacituzumab govitecan vs. chemotherapy for PFS; change in cost and change in QALYs represent the results of sacituzumab govitecan minus chemotherapy.

## Discussion

It is the purpose of this study to satisfy the unmet require for an economic evaluation of SG for the therapy of HR+/HER2- metastatic BC. As a result of this study, it was found that SG was related to an incremental survival of 0.217 QALYs and an incremental cost of $132,689, resulting in ICER of $612,772/QALY, as compared with chemotherapy. The model results were most sensitive to the HRs for OS, average body weight, HRs for PFS, and costs of SG, according to one-way sensitivity analysis. This suggests that the cost-effectiveness of SG can be determined based on these factors compared with chemotherapy. The cost-effectiveness of SG was demonstrated at a WTP threshold of $150,000/QALY when the price of SG was less than $3.997/mg or the weight of patients was less than 19.88 kg. In accordance with one-way sensitivity analysis and probabilistic sensitivity analysis, the results of this model appear to be robust. We found that SG was unfavorable for WTP thresholds less than $612,772/QALY for treatment of HR+/HER2- metastatic BC. Since SG treatment was related to negative INHBs and did not have a probability of cost-effectiveness when compared to chemotherapy at a threshold of $150,000/QALY in all subgroups compared to chemotherapy.

Based on the results of the one-way sensitivity analysis, it was suggested that the HR for OS and PFS was the sensitive variable. There was superior clinical efficacy for SG among patients with a favorable prognosis, but no subgroup analysis revealed that SG achieved cost-effectiveness. Thus, the price of SG remains the most sensitive variable and reducing the price of SG was important to increase the feasibility of using SG. In the US, the government announced American Patients First, and aimed to blueprint for cutting drug prices and reducing out-of-pocket payments (51). The availability of innovative treatments requires a significant reduction in price or financial assistance. Because antibody-drug conjugates are expensive to develop, their prices are often high (26, 52, 53). Therefore, it is common to observe that antibody-drug conjugates are not cost-effective, as described in the published literature (54, 55).

It is critical to highlight the strengths of this study. First, this analysis is the first to synthesize the latest clinical trial in an economic model method in order to evaluate the economic outcomes of SG treatment of HR+/HER2- metastatic BC. Antibody-drug conjugate with an SN-38 payload targeting Trop 2 is a popular option for the therapy of metastatic BC (23, 56). To our knowledge, there is limited data regarding the economic impact of antibody-drug conjugate treatment for metastatic BC. Second, as part of the present study, 22 subgroups defined by the TROPiCS-02 trial were examined in order to determine their economic outcomes. Physicians, patients, and policy makers may benefit from economic information regarding subgroups. The effectiveness of SG treatment needs to be confirmed by further investigation.

Our study has several limitations. First, there are no head-to-head studies for other antibody-drug conjugates, such as trastuzumab-emtansine and trastuzumab-deruxtecan, which have shown benefits for patients with previously treated metastatic BC (57, 58). When head-to-head data becomes available, the current study should be updated. Second, by fitting parametric distributions to the Kaplan-Meier curves, we used the PFS and OS curves reported in the TROPiCS-02 trial, health benefits beyond observation time were assumed. Third, we were unable to take into account the costs associated with follow-up because time series data were not available. Except for the costs of SG, our sensitivity analysis revealed that cost inputs have a limited influence on model outputs. Fourth, the economic results associated with SG may have been overestimated due to the exclusion of costs related to grade 1 or grade 2 AEs. According to the results of the one-way sensitivity analysis, the costs related to AEs were likely to be minor, suggesting that this limitation is not a major concern. It is important to note that the findings of this study are consistent with general clinical practice for the therapy of HR+/HER2- metastatic BC, making them a valuable resource for physicians and policy makers.

## Conclusions

For patients with previously treated HR+/HER2- metastatic BC, SG was unlikely to be a cost-effective therapeutic option. The economic outcomes of treatments can be improved by tailoring them based on the characteristics of the individual patient. The reduction of the cost of SG may result in favorable economic outcomes. The findings of this study may assist clinicians in making optimal treatment choices for patients with HR+/HER2- metastatic BC.

## Data availability statement

The original contributions presented in the study are included in the article/**Supplementary Material**. Further inquiries can be directed to the corresponding author.

## Author contributions

DS: Conceptualization, data interpretation, methodology, formal analysis, Software. YL: Critical revision of the manuscript, validation, data interpretation, formal analysis. XL: Data curation, revision, validation. LC: Conceptualization, methodology, funding acquisition, supervision. All authors contributed to the article and approved the submitted version.
