# Supplementary material for: Cost-effectiveness of sacituzumab govitecan in hormone receptor-positive/human epidermal growth factor receptor 2-negative metastatic breast cancer
Source: Front Oncol. 2023 May 12;13:1162360. doi: 10.3389/fonc.2023.1162360 (PMC10213649; doi:10.3389/fonc.2023.1162360)
Supplement: Supplementary file 1 [file DataSheet_1.docx]

**Supplementary Content**

**Supplementary Figure 1.** Model Fitting Analysis

**Supplementary Figure 2.** Tornado Diagram of One-Way Sensitivity Analyses

**Supplementary Figure 3.** Impacts of Key Factors on Incremental Cost-effectiveness Ratio

**Supplementary Table 1.** Akaike Information Criterion and Bayesian Information Criterion Values from Each Survival Model

**Supplementary Table 2.** Associated Costs and Disutility of Grade ≥ 3 Treatment-Related Adverse Events

**Supplementary Figure 1.** Model Fitting Analysis

To obtain the best model fit, the following investigations were carried out using sacituzumab govitecan or chemotherapy as the model fit baseline, respectively. Based on values of AIC and BIC (Supplementary Table 1), Log-logistic was used to fit the OS K-M curves of sacituzumab govitecan and chemotherapy. Lognormal was used to fit the PFS K-M curves of sacituzumab govitecan and chemotherapy.

(A) Model-fitted versus original K-M curves for sacituzumab govitecan.


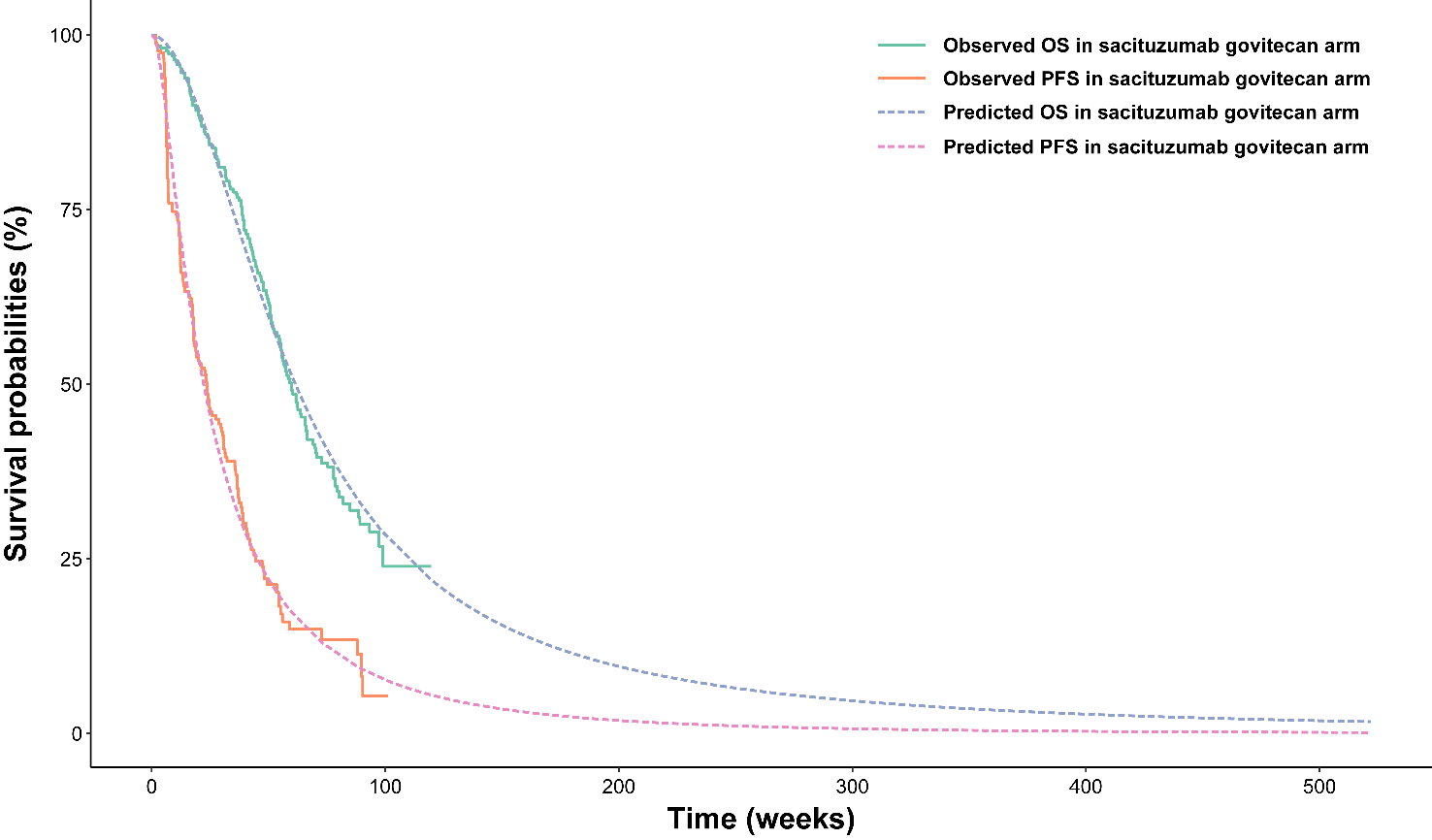


(B) Model-fitted versus original K-M curves for chemotherapy.


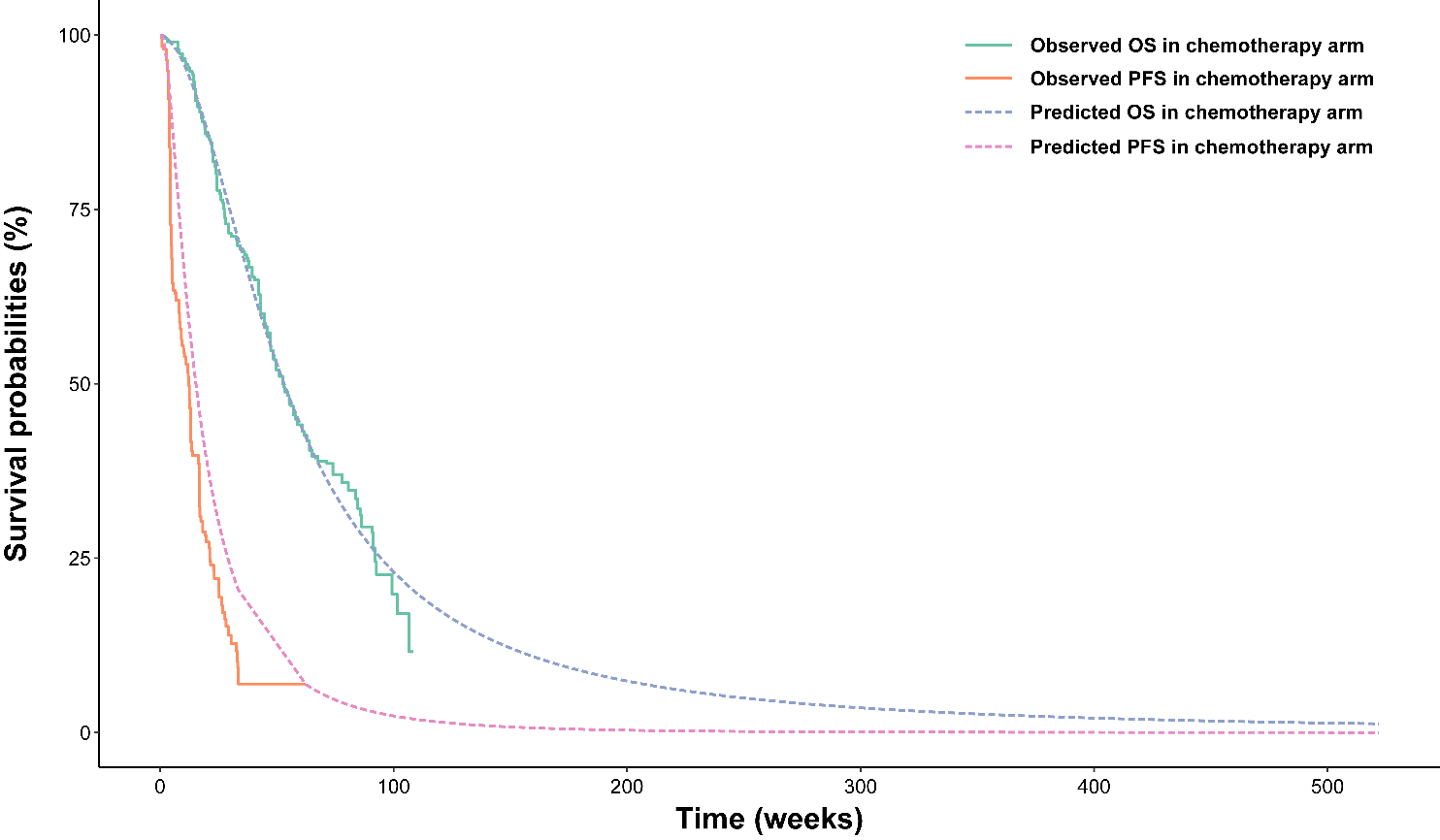


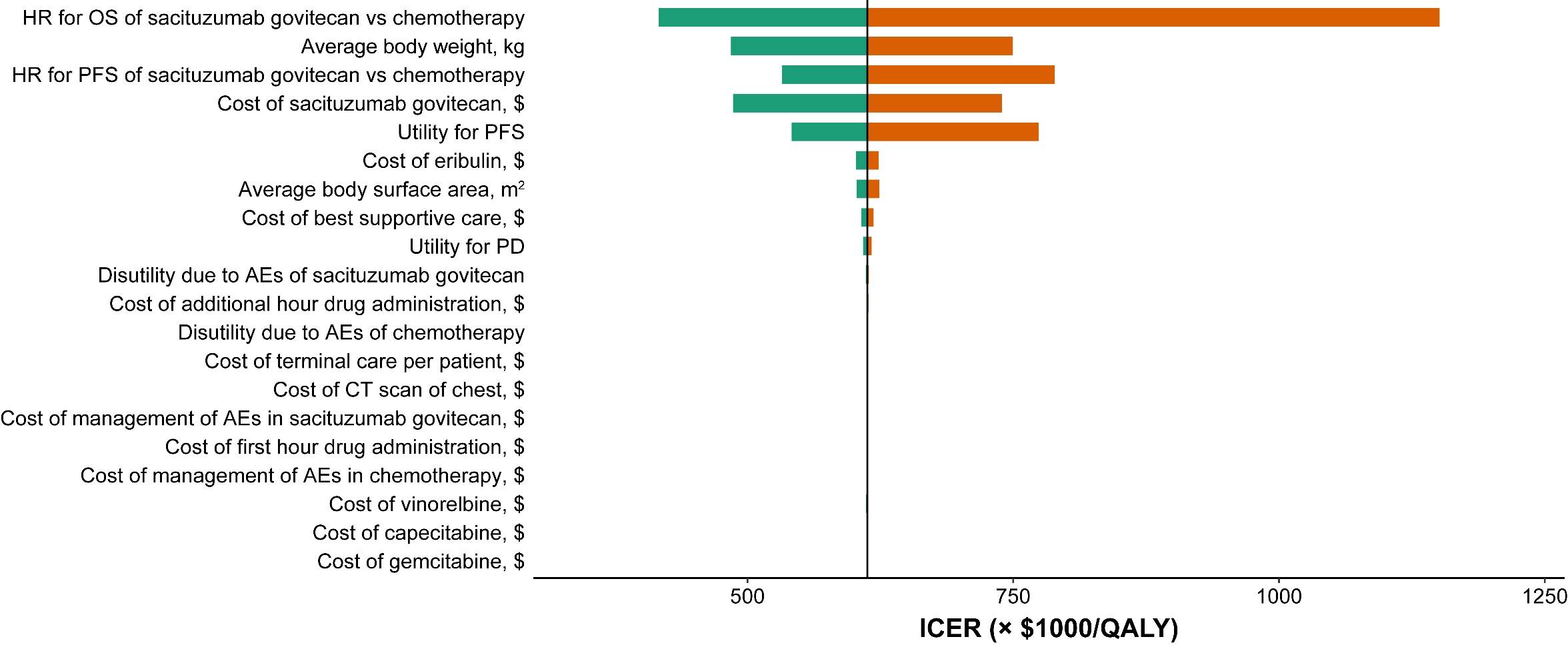


**Supplementary Figure 2.** Tornado Diagram of One-Way Sensitivity Analyses. OS, overall survival; HR, hazard ratio; PD, progressed disease; PFS, progression-free survival; AEs, adverse events.

**Supplementary Figure 3.** Impacts of Key Factors on Incremental Cost-effectiveness Ratio

The diagrams show the impacts of key factors on the incremental cost-effectiveness ratio (sacituzumab govitecan versus chemotherapy) for the treatment of hormone receptor-positive (HR+)/human epidermal receptor 2-negative (HER2-) metastatic breast cancer. (A) represents the impacts of the cost of sacituzumab govitecan; (B) represents the weight of patients. ICER: Incremental cost-effectiveness ratio; QALY: Quality-adjusted life year.

(A) represents the impacts of the cost of sacituzumab govitecan


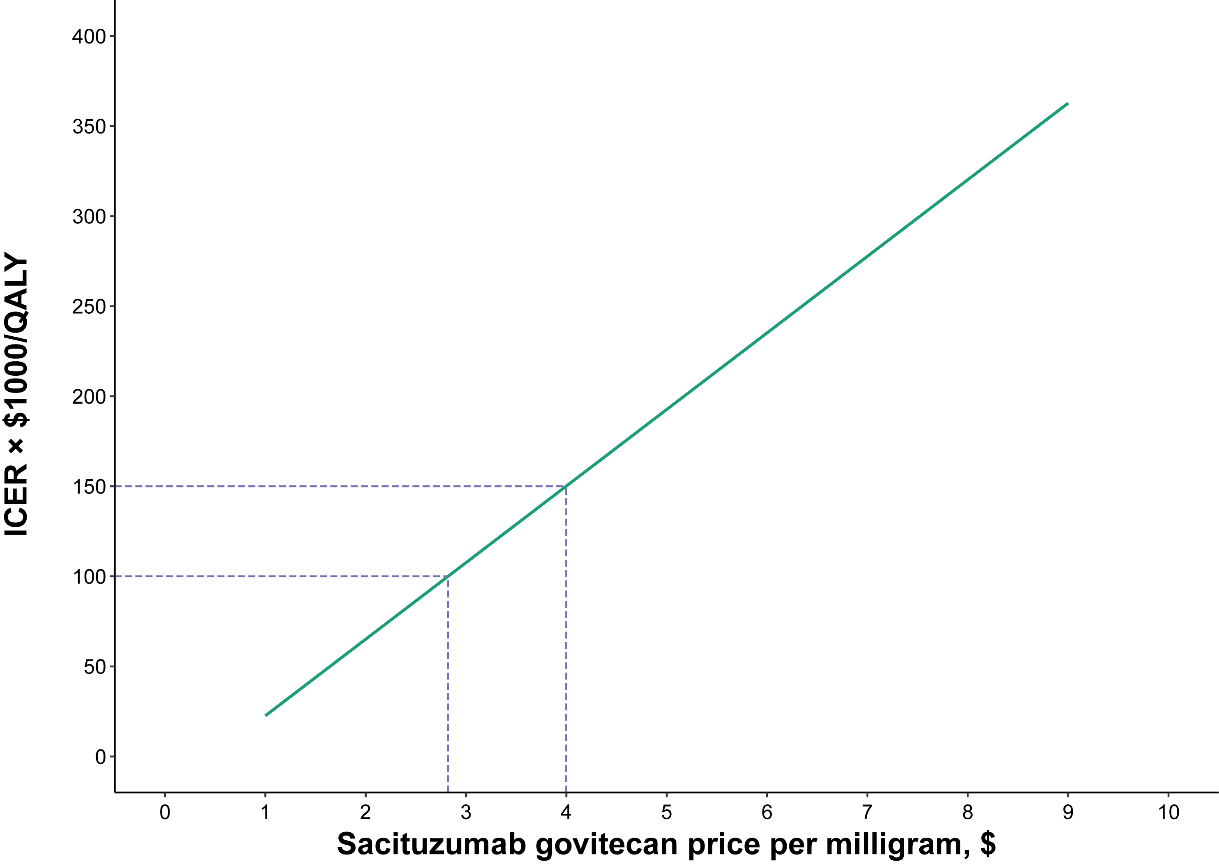


(B) represents the impacts of the weight of patients


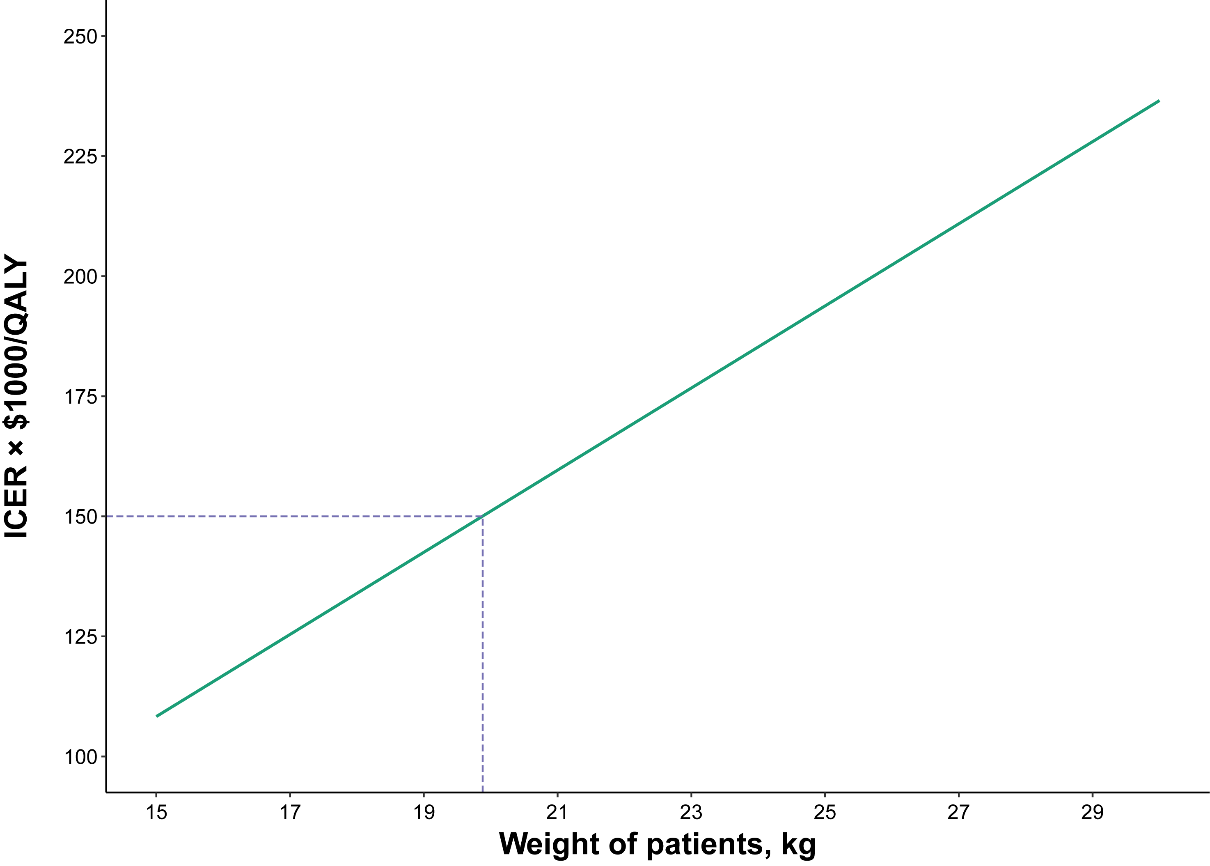


**Supplementary Table 1.** Akaike Information Criterion and Bayesian Information Criterion Values from Each Survival Model. AIC, Akaike information criterion; BIC, Bayesian Information Criterion; OS, overall survival; PFS, progression-free survival.

| **Strategies** | **Distributions** | **Parameters** | **est** | **se** | **L95%** | **U95%** | **AIC** | **BIC** |
| --- | --- | --- | --- | --- | --- | --- | --- | --- |
| **Results of OS** | | | | | | | | |
| **Sacituzumab govitecan** | Exponential | rate | 0.0110 | 0.0009 | 0.0094 | 0.0129 | 1687.754 | 1691.359 |
|  | WeibullPH | shape | 1.5123 | 0.1025 | 1.3242 | 1.7272 | 1659.198 | 1666.41 |
|  |  | scale | 0.0013 | 0.0006 | 0.0006 | 0.0031 |  |  |
|  | Gamma | shape | 1.8143 | 0.1820 | 1.4905 | 2.2084 | 1658.845 | 1666.057 |
|  |  | rate | 0.0239 | 0.0033 | 0.0183 | 0.0312 |  |  |
|  | Lognormal | meanlog | 4.1269 | 0.0714 | 3.9869 | 4.2670 | 1675.378 | 1682.589 |
|  |  | sdlog | 1.0039 | 0.0603 | 0.8924 | 1.1293 |  |  |
|  | Gompertz | shape | 0.0134 | 0.0029 | 0.0078 | 0.0191 | 1669.328 | 1676.539 |
|  |  | rate | 0.0066 | 0.0010 | 0.0050 | 0.0088 |  |  |
|  | Log-logistic | shape | 1.9025 | 0.1311 | 1.6622 | 2.1776 | **1658.721** | **1665.933** |
|  |  | scale | 61.5452 | 3.7332 | 54.6466 | 69.3148 |  |  |
|  | Generalized gamma | mu | 4.3612 | 0.0787 | 4.2070 | 4.5154 | 1660.459 | 1671.276 |
|  |  | sigma | 0.6958 | 0.0820 | 0.5523 | 0.8766 |  |  |
|  |  | Q | 0.8791 | 0.2293 | 0.4297 | 1.3285 |  |  |
| **Chemotherapy** | Exponential | rate | 0.0127 | 0.0010 | 0.0108 | 0.0149 | 1590.303 | 1593.905 |
|  | WeibullPH | shape | 1.5107 | 0.1017 | 1.3240 | 1.7237 | 1560.834 | 1568.038 |
|  |  | scale | 0.0016 | 0.0007 | 0.0007 | 0.0037 |  |  |
|  | Gamma | shape | 1.8686 | 0.1892 | 1.5323 | 2.2787 | 1558.923 | 1566.127 |
|  |  | rate | 0.0283 | 0.0038 | 0.0217 | 0.0370 |  |  |
|  | Lognormal | meanlog | 3.9698 | 0.0685 | 3.8356 | 4.1040 | 1565.384 | 1572.588 |
|  |  | sdlog | 0.9427 | 0.0569 | 0.8376 | 1.0611 |  |  |
|  | Gompertz | shape | 0.0146 | 0.0031 | 0.0084 | 0.0207 | 1572.277 | 1579.481 |
|  |  | rate | 0.0077 | 0.0011 | 0.0057 | 0.0102 |  |  |
|  | Log-logistic | shape | 1.9082 | 0.1294 | 1.6707 | 2.1794 | **1560.008** | **1567.212** |
|  |  | scale | 53.1343 | 3.3383 | 46.9781 | 60.0972 |  |  |
|  | Generalized gamma | mu | 4.1532 | 0.0923 | 3.9722 | 4.3341 | 1560.65 | 1571.456 |
|  |  | sigma | 0.7712 | 0.0846 | 0.6220 | 0.9563 |  |  |
|  |  | Q | 0.6016 | 0.2452 | 0.1210 | 1.0822 |  |  |
| **Results of PFS** | | | | | | | | |
| **Sacituzumab govitecan** | Exponential | rate | 0.0286 | 0.0022 | 0.0246 | 0.0332 | 1559.612 | 1563.217 |
|  | WeibullPH | shape | 1.1429 | 0.0667 | 1.0193 | 1.2815 | 1556.73 | 1563.942 |
|  |  | scale | 0.0174 | 0.0043 | 0.0107 | 0.0282 |  |  |
|  | Gamma | shape | 1.3154 | 0.1188 | 1.1020 | 1.5700 | 1552.981 | 1560.192 |
|  |  | rate | 0.0402 | 0.0051 | 0.0313 | 0.0516 |  |  |
|  | Lognormal | meanlog | 3.1013 | 0.0724 | 2.9593 | 3.2433 | **1533.99** | **1541.202** |
|  |  | sdlog | 1.0541 | 0.0582 | 0.9460 | 1.1746 |  |  |
|  | Gompertz | shape | 0.0005 | 0.0041 | -0.0074 | 0.0085 | 1561.596 | 1568.808 |
|  |  | rate | 0.0283 | 0.0032 | 0.0227 | 0.0353 |  |  |
|  | Log-logistic | shape | 1.5921 | 0.0977 | 1.4116 | 1.7956 | 1542.767 | 1549.978 |
|  |  | scale | 22.1159 | 1.6296 | 19.1419 | 25.5520 |  |  |
|  | Generalized gamma | mu | 2.9503 | 0.1444 | 2.6674 | 3.2332 | 1534.49 | 1545.307 |
|  |  | sigma | 1.0810 | 0.0615 | 0.9670 | 1.2085 |  |  |
|  |  | Q | -0.3267 | 0.2659 | -0.8480 | 0.1945 |  |  |
| **Chemotherapy** | Exponential | rate | 0.0421 | 0.0032 | 0.0362 | 0.0490 | 1410.561 | 1414.163 |
|  | WeibullPH | shape | 1.2145 | 0.0683 | 1.0878 | 1.3561 | 1401.851 | 1409.055 |
|  |  | scale | 0.0216 | 0.0050 | 0.0137 | 0.0339 |  |  |
|  | Gamma | shape | 1.4963 | 0.1362 | 1.2518 | 1.7885 | 1394.759 | 1401.963 |
|  |  | rate | 0.0688 | 0.0085 | 0.0540 | 0.0877 |  |  |
|  | Lognormal | meanlog | 2.7297 | 0.0659 | 2.6005 | 2.8589 | **1372.462** | **1379.666** |
|  |  | sdlog | 0.9475 | 0.0519 | 0.8511 | 1.0548 |  |  |
|  | Gompertz | shape | 0.0021 | 0.0053 | -0.0083 | 0.0125 | 1412.404 | 1419.609 |
|  |  | rate | 0.0409 | 0.0044 | 0.0330 | 0.0505 |  |  |
|  | Log-logistic | shape | 1.7858 | 0.1089 | 1.5846 | 2.0125 | 1379.352 | 1386.556 |
|  |  | scale | 15.0525 | 1.0138 | 13.1910 | 17.1767 |  |  |
|  | Generalized gamma | mu | 2.6196 | 0.1129 | 2.3982 | 2.8409 | 1373.049 | 1383.856 |
|  |  | sigma | 0.9651 | 0.0544 | 0.8642 | 1.0779 |  |  |
|  |  | Q | -0.2594 | 0.2149 | -0.6806 | 0.1618 |  |  |

**Supplementary Table 2.** Associated Costs and Disutility of Grade ≥ 3 Treatment-Related Adverse Events

| **Adverse Event^a^** | **No. of patients (%)^b^** | **Costs in 2022 USD** | **Reference** | **Disutility** | **Reference** |
| --- | --- | --- | --- | --- | --- |
| **Sacituzumab govitecan** | |  |  |  |  |
| Febrile neutropenia | 140 (52%) | 9649 | (1) | 0.030 | (1) |
| Anemia | 17 (6%) | 15196 | (2) | 0.073 | (2) |
| Leukopenia | 23 (9%) | 4934 | (1) | 0.003 | (1) |
| Nausea/vomiting | 4 (1%) | 8019 | (3) | 0.088 | (3) |
| Diarrhea | 25 (9%) | 7456 | (3) | 0.103 | (3) |
| Fatigue | 15 (6%) | 1172 | (3) | 0.099 | (3) |
| **Total^c^** |  | 7309 |  | 0.037 |  |
| **Chemotherapy** |  |  |  |  |  |
| Febrile neutropenia | 105 (42%) | 9649 | (1) | 0.030 | (1) |
| Anemia | 8 (3%) | 15196 | (2) | 0.073 | (2) |
| Leukopenia | 13 (5%) | 4934 | (1) | 0.003 | (1) |
| Nausea/vomiting | 11 (4%) | 8019 | (3) | 0.088 | (3) |
| Diarrhea | 3 (1%) | 7456 | (3) | 0.103 | (3) |
| Fatigue | 6 (2%) | 1172 | (3) | 0.099 | (3) |
| **Total^c^** |  | 5287 |  | 0.023 |  |

^a^Our analysis only included and evaluated grade ≥ 3 treatment-related adverse events.

^b^Number within treatment arm: sacituzumab govitecan (N = 268), chemotherapy (N = 249).

^c^Calculated as an average cost of toxicity using the weighted frequency of occurrence. This value was used in the base-case model.

**References**

1. Jeong E, Wang C, Wilson L, Zhong L. Cost-Effectiveness of Adding Ribociclib to Endocrine Therapy for Patients with Hr-Positive, Her2-Negative Advanced Breast Cancer among Premenopausal or Perimenopausal Women. *Front Oncol* (2021) 11:658054. Epub 2021/05/25. doi: 10.3389/fonc.2021.658054.

2. Wang H, Wang Y, Gong R, Geng Y, Li L. Cost-Effectiveness of Pertuzumab and Trastuzumab as a First-Line Treatment of Her2-Positive Metastatic Breast Cancer in China. *Ann Palliat Med* (2021) 10(11):11382-93. Epub 2021/12/08. doi: 10.21037/apm-21-2412.

3. Diaby V, Adunlin G, Ali AA, Zeichner SB, de Lima Lopes G, Kohn CG, et al. Cost-Effectiveness Analysis of 1st through 3rd Line Sequential Targeted Therapy in Her2-Positive Metastatic Breast Cancer in the United States. *Breast Cancer Res Treat* (2016) 160(1):187-96. Epub 2016/09/23. doi: 10.1007/s10549-016-3978-6.
